# Factors associated with oral HPV infection and clearance in people living with HIV and populations at increased risk of HIV acquisition: a systematic review and meta-analysis

**DOI:** 10.3389/fcimb.2026.1963308

**Published:** 2026-10-29

**Authors:** Mengjie Lin, Haibo Jiang, Lixia Ye, Shiwen Tan, Kun Chu, Zehao Ye, Pingping Xu, Yiya Wang, Yu Wang, Yi Chen, Zhixin Zhu

**Affiliations:** 1 Department of Implantology, Ningbo Stomatology Hospital, Ningbo, Zhejiang, China; 2 Ningbo Municipal Center for Disease Control and Prevention, Ningbo, China; 3 The Professional Committee for the Digital Prevention and Control of AIDS and STDs, Zhejiang Provincial Association for AIDS and STD Prevention and Control, Hangzhou, China; 4 No. 906 Hospital of the Joint Logistics Support Force of the Chinese People’s Liberation Army, Ningbo, China

**Keywords:** HIV, HPV clearance, meta-analysis, oral human papillomavirus, risk factors

## Abstract

**Background:**

Oral human papillomavirus (HPV) infection precedes most HPV-related oropharyngeal cancers, but determinants of oral HPV prevalence and incidence in people living with HIV (PLWH) and populations at increased risk of HIV acquisition remain unclear.

**Methods:**

PubMed, Embase, and the Cochrane Library were searched through July 10, 2026, for observational studies of laboratory-confirmed oral HPV prevalence, incidence, or clearance in adults with or at risk for HIV. Adjusted estimates were pooled by random-effects meta-analysis and assessed with GRADE.

**Results:**

Twenty-seven studies with a cumulative reported sample size of 13,625 participants were included, of which 25 contributed to the meta-analysis. For oral HPV prevalence, smoking (n = 8; OR, 2.11; 95% CI, 1.58–2.83), HIV infection (n = 6; OR, 2.44; 95% CI, 1.69–3.52), and lower CD4 count (n = 8; OR, 3.02; 95% CI, 2.10–4.35) were significantly associated with higher odds, whereas alcohol, recent oral-sex partners, and HIV viral load were not. For oral HPV incidence, more recent oral-sex partners (n = 3; HR, 1.88; 95% CI, 1.41–2.49), more lifetime oral-sex partners (n = 3; HR, 2.19; 95% CI, 1.03–4.68), HIV infection (n = 2; HR, 1.90; 95% CI, 1.20–3.02), lower CD4 count (n = 4; HR, 1.85; 95% CI, 1.43–2.40), and higher HIV viral load (n = 2; HR, 2.51; 95% CI, 1.28–4.93) were significantly associated with increased incidence, whereas smoking and alcohol were not. HIV infection showed a borderline association with reduced clearance (n = 3; HR, 0.43; 95% CI, 0.19–1.00). Certainty of evidence ranged from low to very low.

**Conclusions:**

Smoking was associated with oral HPV prevalence, whereas greater oral-sex exposure and higher HIV viral load were more clearly associated with incidence. HIV infection and lower CD4 counts were associated with both outcomes, while evidence for clearance remained inconclusive. Further prospective studies are needed.

## 1 Background

Human papillomavirus (HPV)-associated oropharyngeal squamous cell carcinoma is an increasing component of the head and neck cancer burden, particularly in many high-income settings, with HPV16 causing most HPV-driven tumors (Lechner et al., 2022; Wierzbicka et al., 2023; Chen, 2024). Persistent oncogenic oral HPV infection is the key detectable precursor state, although progression from infection to malignancy is uncommon and may take years, making persistence rather than transient detection the more biologically relevant natural-history endpoint (D'Souza et al., 2024; Lim and D'Silva, 2024).

People living with HIV (PLWH) experience a disproportionate burden of HPV-associated cancers, including oropharyngeal cancer, compared with the general population even in the antiretroviral therapy era (Tisler et al., 2024; Bandala-Jacques et al., 2025). Consistent with this disparity, oral high-risk HPV and HPV16 are more prevalent in PLWH than in HIV-negative populations (Dube Mandishora et al., 2025). Importantly, oral HPV prevalence, incidence, and clearance represent distinct aspects of the natural history of infection. While prevalence reflects both the acquisition and duration of infection, incidence captures newly detected infections during follow-up, and clearance reflects the resolution of detectable infection (D'Souza et al., 2024; Dube Mandishora et al., 2024; D'Souza et al., 2025; Dickey et al., 2025).

The excess oral HPV burden in PLWH is biologically plausible because impaired cell-mediated immunity may weaken control of HPV-infected epithelium and facilitate HPV acquisition and persistence (D'Souza et al., 2025). Longitudinal data show higher oral HPV prevalence and greater persistence among HIV-positive adults, particularly those with a history of poorly controlled HIV (Riddell et al., 2022). Lower CD4 cell counts and detectable HIV viremia are also associated with oral HPV, suggesting that immune competence and virologic control shape susceptibility (Carnalla et al., 2023; Sotelo and Deluca, 2023). Studies from North America, Latin America, and Africa further indicate that HIV-related differences occur across diverse populations and HPV genotype distributions despite differences in age and HIV treatment context (Riddell et al., 2022; Beltrame et al., 2026a; Nowak et al., 2026).

Behavioral exposures may compound immunologic vulnerability because oral HPV is transmitted through intimate mucosal contact and is repeatedly associated with sexual exposure and tobacco use (Giuliani et al., 2021). Recent oral-sex partners and cumulative lifetime oral-sex partners may capture distinct dimensions of exposure, including short-term acquisition and cumulative contact (Giuliani et al., 2021; Dube Mandishora et al., 2024). Tobacco smoking is particularly relevant in PLWH, and HIV may amplify the association between smoking and oral high-risk HPV detection (Alli et al., 2020). By contrast, the relationship between alcohol consumption and oral HPV remains inconsistent, with recent HIV-focused studies reporting null or imprecise associations (Sotelo and Deluca, 2023; Beltrame et al., 2026a).

Evidence regarding the determinants of oral HPV infection in PLWH and populations at increased risk of HIV acquisition remains fragmented and inconsistent. In particular, associations with smoking, sexual exposure, and HIV-related immunologic or virologic factors have not been consistently reproduced (Beachler et al., 2015; Alli et al., 2020; Carnalla et al., 2023), while alcohol consumption has generally shown null or imprecise associations (Sotelo and Deluca, 2023; Beltrame et al., 2026b). Previous systematic reviews have primarily characterized the prevalence and burden of oral HPV among PLWH rather than quantitatively evaluating its determinants (Dube Mandishora et al., 2025). Although longitudinal studies have investigated oral HPV acquisition and clearance (Beachler et al., 2015; Dube Mandishora et al., 2024; D'Souza et al., 2025), the factors associated with prevalent and incident infections remain insufficiently synthesized and compared. Therefore, the present study aimed to systematically evaluate the associations of behavioral and HIV-related factors with oral HPV prevalence, incidence, and clearance separately, while exploring potential sources of between-study heterogeneity.

## 2 Methods

The protocol was registered with PROSPERO (CRD420261446947), and the review was conducted in accordance with the PRISMA guidelines (Page et al., 2021).

### 2.1 Search strategy

PubMed, Embase, and the Cochrane Library were searched from inception to July 10, 2026, using terms related to oral HPV infection, clearance, HIV, and associated factors. The full search strategy is provided in **Supplementary Table S1**. Reference lists of included studies and relevant reviews were also screened, with no additional eligible studies identified.

### 2.2 Study selection

Two reviewers independently screened the titles and abstracts of all retrieved records and subsequently assessed the full texts of potentially eligible studies. Disagreements were resolved through discussion or consultation with a third reviewer. Study selection was conducted according to predefined eligibility criteria.

We included full-length, peer-reviewed observational studies involving people aged 14 years or older who were living with HIV or belonged to populations at increased risk of HIV infection. Eligible studies evaluated factors associated with oral HPV infection, persistence, or clearance. Factors of interest primarily included lifestyle-related factors, such as smoking and alcohol consumption; sexual behavior-related factors, particularly the numbers of recent and lifetime oral sex partners; and HIV-related factors, including HIV status, CD4 cell count, and HIV viral load. When multiple studies reported the same population and exposure variable, only the most recent study or the study with the largest sample size was included. Oral HPV infection was required to be determined using laboratory testing of oral specimens. We excluded studies involving pediatric populations, non-observational designs, unrelated exposures or outcomes, or populations not relevant to HIV or HIV-risk settings. Reviews, meta-analyses, case reports, letters, editorials, conference abstracts, and studies with insufficient data were also excluded.

### 2.3 Data extraction

Two reviewers (M.J.L. and Z.X.Z.) independently extracted data from the included studies using a standardized data extraction form. The extracted information was verified by a third reviewer (Y.C.), and any discrepancies were resolved through discussion and consensus. The following data were collected: first author and publication year, country, study design, study population, sample size, age, proportion of men who have sex with men, proportion of participants living with HIV, investigated factors, and study outcomes. Only the most fully covariate-adjusted effect estimates were extracted. When multiple adjusted estimates were reported, effect measures were prioritized in the following order: adjusted hazard ratio (aHR), adjusted risk ratio (aRR), adjusted odds ratio (aOR), and adjusted prevalence ratio (aPR).

### 2.4 Risk of bias assessment

Two reviewers (M.J.L. and Z.X.Z.) independently evaluated the methodological quality of the included observational studies using the Newcastle-Ottawa Scale (NOS) (Cook and Reed, 2015). Any discrepancies were discussed and, when necessary, adjudicated by a third reviewer (Y.C.). The NOS assesses nonrandomized studies across three broad domains: selection of participants, comparability of study groups, and ascertainment of exposure or outcomes. Depending on the study design, the assessment considered the representativeness of the study population, participant selection, exposure measurement, confirmation that the outcome was absent at baseline, control of potential confounders, outcome assessment, follow-up duration, and completeness of follow-up. One point was assigned for each criterion judged to be adequately fulfilled, yielding a maximum score of 9. Studies scoring 7-9, 4-6, and 0–3 points were classified as having low, moderate, and high risk of bias, respectively.

### 2.5 Outcomes

The primary outcomes were the prevalence and incidence of laboratory-confirmed oral HPV infection, including high-risk and low-risk HPV types and HPV genotypes belonging to the alpha, beta, and gamma genera. High-risk HPV (HR-HPV) infection was defined as infection with HPV types 16, 18, 31, 33, 35, 39, 45, 51, 52, 56, 58, 59, 66, or 68. The secondary outcome was HPV clearance. Studies that reported effect estimates for HPV persistence rather than clearance were included in the systematic review but excluded from the meta-analysis.

### 2.6 Statistical analyses

All analyses were conducted using R version 4.5.0 (R Foundation for Statistical Computing) with the *meta* package (Balduzzi et al., 2019). When at least two studies reported comparable data, the maximally adjusted effect estimates were pooled using a random-effects model and reported with 95% confidence intervals. Odds ratios (ORs) were used for prevalence, and hazard ratios (HRs) were used for incidence and clearance. For studies reporting multiple exposure categories, only effect estimates comparing the highest versus the lowest exposure levels were included in the meta-analysis. Statistical heterogeneity was assessed using the I² statistic, with values of approximately 25%, 50%, and 75% representing low, moderate, and high heterogeneity, respectively. Potential sources of heterogeneity were explored through subgroup analyses according to HPV type, HIV status, and geographical region. Meta-regression was further performed to determine whether differences between subgroups were statistically significant. Publication bias was assessed by visual inspection of funnel plots and further evaluated using Egger’s and Begg’s tests. A 2-sided *P* value of ≤0.05 was considered statistically significant.

### 2.7 Overall quality of evidence

The overall certainty of the pooled evidence for each outcome was assessed using the Grading of Recommendations Assessment, Development and Evaluation (GRADE) framework (Guyatt et al., 2008). The assessment considered study design, risk of bias, inconsistency, indirectness, imprecision, and publication bias. The certainty of evidence was classified as high, moderate, low, or very low.

## 3 Results

The PRISMA flowchart outlining the study selection process is presented in **Figure 1**. The search of three databases identified 1,340 records, with an additional 7 records obtained from other sources. After removing 424 duplicates, 923 records were screened, of which 506 were excluded. Among 417 full-text articles assessed for eligibility, 390 were excluded. Finally, 27 studies were included in both the systematic review and 25 were included in the final meta-analysis (Coutlée et al., 1997; D'Souza et al., 2007; Read et al., 2012; Beachler et al., 2013; Mooij et al., 2013; Darwich et al., 2014; Gaester et al., 2014; Mooij et al., 2014; Beachler et al., 2015; Muller et al., 2015; Robbins et al., 2015; van Aar et al., 2014; Oliver et al., 2018; Vergori et al., 2018; Alli et al., 2020; Giuliani et al., 2020; Pérez-Quintanilla et al., 2020; D'Souza et al., 2020; Giuliani et al., 2021; Riddell et al., 2022; Carnalla et al., 2023; Sotelo and Deluca, 2023; D'Souza et al., 2024; D'Souza et al., 2025; Hidalgo-Tenorio et al., 2025; Beltrame et al., 2026b; Nowak et al., 2026).

**Figure 1:**
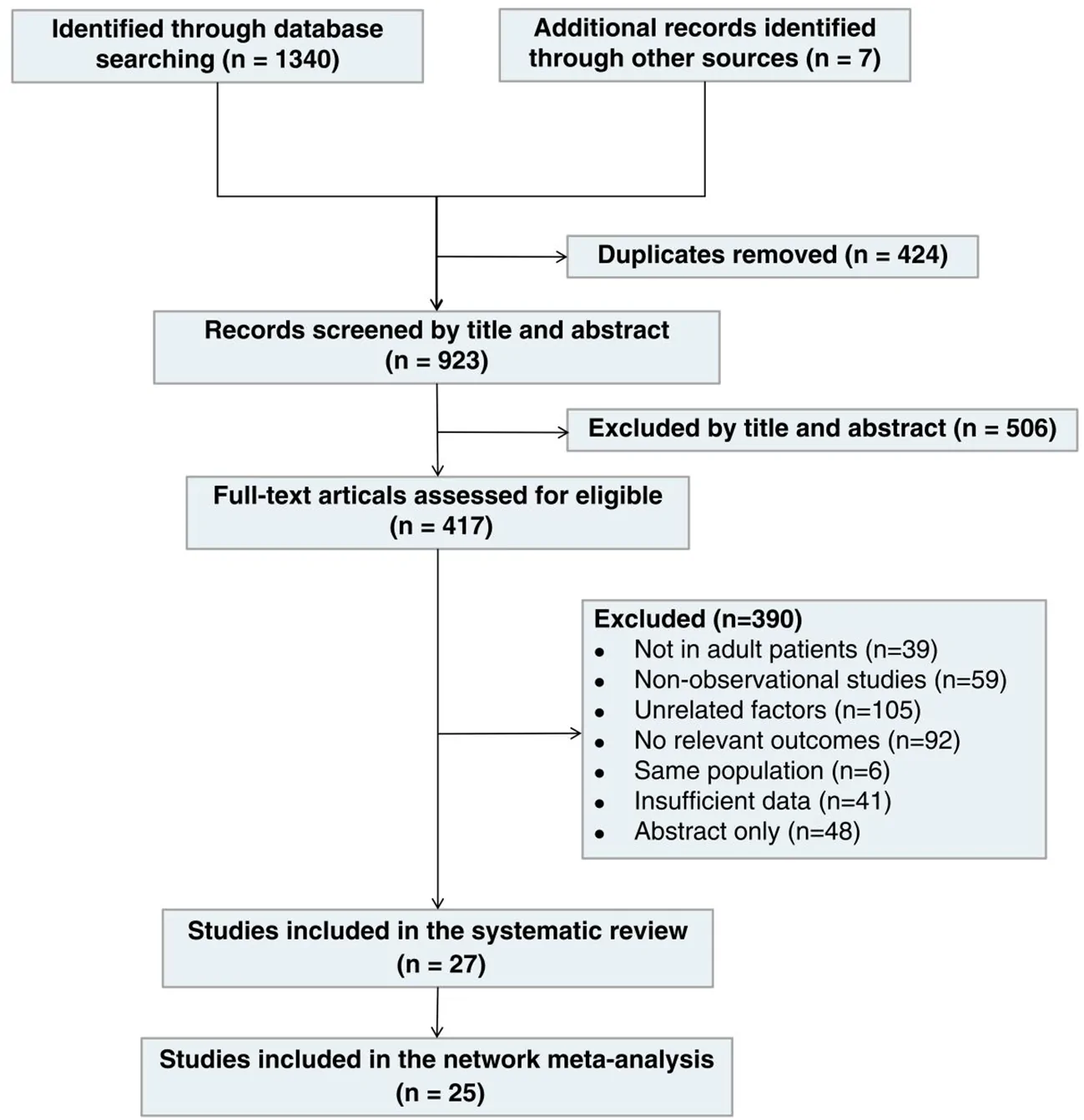
PRISMA flow diagram of the study selection process.

### 3.1 Study characteristics

The characteristics of the included studies are summarized in **Table 1**. A total of 27 observational studies were included, comprising 12 prospective cohort studies and 15 cross-sectional studies, with a cumulative reported sample size of 13,625 participants. Participants were predominantly young to middle-aged adults, with reported mean or median ages generally ranging from the late 30s to early 50s. Approximately 70% of participants in studies reporting MSM status were MSM, while approximately 62% of the reported study participants were living with HIV. The studies were most commonly conducted in the United States (n = 11), Italy (n = 3), and the Netherlands (n = 3). Three studies included participants from Mexico, including one multicountry study. According to the Newcastle-Ottawa Scale, 14 studies (51.9%) were classified as having a low risk of bias, 11 (40.7%) as having a moderate risk, and 2 (7.4%) as having a high risk. Among the 15 cross-sectional studies, 3 were classified as having a low risk of bias, 10 as moderate risk, and 2 as high risk. Detailed risk-of-bias assessments are provided in **Supplementary Tables S2** and **S3**.

**Table 1:** Characteristics of included studies.

Author-year ^ref.^	Country	Study type	Population	Number of participants	Age (year)	MSM (%)	HIV-positive (%)	Study factors	Outcomes
Alli 2020 (Alli et al., 2020)	US	Cross-sectional	Outpatients in the MuCOSAL study	295	Mean 52.1 (SD 11.8)	7.5%	57.3%	Smoking; HIV infection	Prevalence (α-HPV, HR-HPV)
Beachler 2013 (Beachler et al., 2013)	US	Prospective cohort	HIV-positive clinic patients	404	Median 46.4 (IQR 42.1-51.0)	18.0%	100%	Smoking; recent oral-sex partners; lifetime oral-sex partners; nadir CD4 count; current CD4 count	Incidence
Beachler 2015 (Beachler et al., 2015)	US	Prospective cohort	POPS participants from MACS/WIHS	1,230	Median 49.7 (IQR 43.0-56.2)	45.0%	61.9%	Smoking; alcohol use; recent oral-sex partners; lifetime oral-sex partners; HIV infection; current CD4 count	Incidence; Clearance
Beltrame 2026 (Beltrame et al., 2026b)	Brazil; Mexico; Puerto Rico	Cross-sectional	Men living with HIV in ULACNet-201	699	≥18	97.7%	100%	Alcohol use	Prevalence (HR-HPV, LR-HPV)
Carnalla 2023 (Carnalla et al., 2023)	Mexico	Cross-sectional	MSM attending two STI clinics	485	≥18	100%	40.4%	CD4 category	Prevalence (HR-HPV)
Coutlée 1997 (Coutlée et al., 1997)	Canada	Cross-sectional	Sexually active STD/HIV and endoscopy-clinic patients	287	Mean 36.7 (SD 9.3; range 16.1-60.3)	61.7%	62.0%	HIV infection	Prevalence
D’Souza 2007 (D'Souza et al., 2007)	US	Prospective cohort	WIHS cohort	182	≥18	0%	67.6%	Smoking; HIV infection; CD4 count; HIV viral load	Persistence
D’Souza 2020 (D'Souza et al., 2020)	US	Prospective cohort	POPS/MOUTH participants with oncogenic oral HPV	447	Median 50.0	59.1%	73.4%	HIV infection; CD4 category	Clearance (oncogenic HPV, HPV16)
D’Souza 2024 (D'Souza et al., 2024)	US	Prospective cohort	POPS/MOUTH participants with oncogenic oral HPV	435	Median 49.6 (IQR 43.3-55.5)	58.4%	73.8%	HIV infection	Clearance (oncogenic HPV)
D’Souza 2025 (D'Souza et al., 2025)	US	Prospective cohort	MWCCS POPS/MOUTH participants	1,587	Median 48.6	47.7%	66.2%	Smoking; recent oral-sex partners; CD4 category	Incidence
Darwich 2014 (Darwich et al., 2014)	Spain	Prospective cohort	CARH-MEN cohort	733	Median 41.0	73.4%	100%	Nadir CD4 count	Prevalence (HPV16/18)
Gaester 2014 (Gaester et al., 2014)	Brazil	Cross-sectional	HIV-positive male outpatients	283	Mean 43.81 (range 22-72)	41.0%	100%	Smoking; alcohol use	Prevalence
Giuliani 2020 (Giuliani et al., 2020)	Italy	Prospective cohort	OHMAR cohort	244	≥18	100%	42.2%	Smoking; recent oral-sex partners; lifetime oral-sex partners; nadir CD4 count	Incidence; Clearance
Giuliani 2021 (Giuliani et al., 2021)	Italy	Cross-sectional	OHMAR cohort	310	Median 43 HIV+; 39 HIV−	100%	37.7%	Smoking; recent receptive oral-sex partners; current CD4 count	Prevalence (α-HPV, LR-HPV)
Hidalgo-Tenorio 2025 (Hidalgo-Tenorio et al., 2025)	Spain	Prospective cohort	PWH in anal/genital cancer-screening programs	276	Mean 45.3	78.3%	100%	Smoking; HIV viral load	Incidence
Mooij 2013 (Mooij et al., 2013)	Netherlands	Cross-sectional	H2M cohort	767	Median 40.1 (IQR 34.8-47.5)	100%	40.9%	Smoking; recent oral-sex partners; HIV infection; HIV viral load	Prevalence (HR-HPV)
Mooij 2014 (Mooij et al., 2014)	Netherlands	Prospective cohort	MSM with baseline and 6-month H2M data	689	Median 40.3 (IQR 34.9-47.5)	100%	40.1%	Smoking; HIV infection; current CD4 count; HIV viral load	Persistence
Muller 2015 (Muller et al., 2015)	US	Cross-sectional	Dental and oral-medicine patients	289	Mean 50.8 (range 21-80)	7.6%	55.7%	Smoking; current CD4 count	Prevalence
Nowak 2026 (Nowak et al., 2026)	Nigeria	Cross-sectional	Men with same-sex sexual contact in TRUST/RV368	584	Median 24 (IQR 22-28)	100%	69.2%	HIV infection	Prevalence (HR-HPV)
Oliver 2018 (Oliver et al., 2018)	US	Cross-sectional	Young MSM recruited from community clinics	922	Range 18-26	100%	9.5%	Smoking; recent oral-sex partners; HIV infection	Prevalence
Pérez-Quintanilla 2020 (Pérez-Quintanilla et al., 2020)	Mexico	Cross-sectional	HIV-positive women attending a specialized clinic	174	Mean 40.6 (SD 12.52)	0%	100%	Smoking; recent oral-sex partners; current CD4 count	Prevalence (HPV16, HPV16/18, HPV66, HR-HPV)
Read 2012 (Read et al., 2012)	Australia	Cross-sectional	MSM attending a sexual-health centre	500	Median 37 (IQR 27-45)	100%	49.8%	Smoking; recent oral-sex partners; lifetime oral-sex partners; HIV infection	Prevalence
Riddell 2022 (Riddell et al., 2022)	US	Prospective cohort	PLWH and HIV-negative controls from five academic centers	443	≥18	NR	55.3%	HIV infection; current CD4 count; HIV viral load	Incidence; Clearance
Robbins 2015 (Robbins et al., 2015)	US	Cross-sectional	ALIVE participants with injection-drug-use history	199	Median 54	5.5%	32.2%	CD4 category	Prevalence
Sotelo 2023 (Sotelo and Deluca, 2023)	Argentina	Cross-sectional	People living with HIV in Corrientes Province	133	Mean 37 (range 18-66)	NR	100%	Smoking; alcohol use; CD4 percentage; HIV viral load	Prevalence (HR-HPV)
van Aar 2014 (van Aar et al., 2014)	Netherlands	Prospective cohort	H2M cohort	723	Median 38 (IQR 34-42) HIV−; 47 (IQR 40-53) HIV+	100%	40.1%	Smoking/cannabis use; recent oral-sex partners; HIV infection; nadir CD4 count	Incidence (HR-HPV); Clearance (HR-HPV)
Vergori 2018 (Vergori et al., 2018)	Italy	Cross-sectional	HIV-positive men in an anal-cancer screening program	305	Median 44.7 (IQR 37.4-51.0)	85.9%	100%	Current CD4 count; HIV viral load	Prevalence

### 3.2 Factors associated with any oral HPV prevalence

For oral HPV prevalence, smoking was associated with significantly increased odds of infection across 8 studies contributing 10 effect estimates (OR, 2.11; 95% CI, 1.58-2.83; I² = 0%) (**Figure 2**). In contrast, alcohol consumption was not significantly associated with oral HPV prevalence across 3 studies (OR, 0.88; 95% CI, 0.64-1.21; I² = 24.5%). A greater number of recent oral-sex partners was associated with higher estimated odds of infection, although the association was not statistically significant and substantial heterogeneity was observed across 4 studies (OR, 1.40; 95% CI, 0.59-3.32; I² = 72.8%). Greater lifetime oral-sex exposure was significantly associated with oral HPV prevalence in the single contributing study (OR, 3.20; 95% CI, 1.41-7.26). Among HIV-related factors, HIV infection was associated with significantly increased odds of prevalent oral HPV infection across 6 studies (OR, 2.44; 95% CI, 1.69-3.52; I² = 33.4%). Similarly, lower CD4 cell counts were associated with approximately threefold higher odds of infection across 8 studies (OR, 3.02; 95% CI, 2.10-4.35; I² = 14.9%). Higher HIV viral load was associated with increased estimated odds of infection, but the association did not reach statistical significance across 3 studies (OR, 1.59; 95% CI, 0.93-2.74; I² = 21.0%).

**Figure 2:**
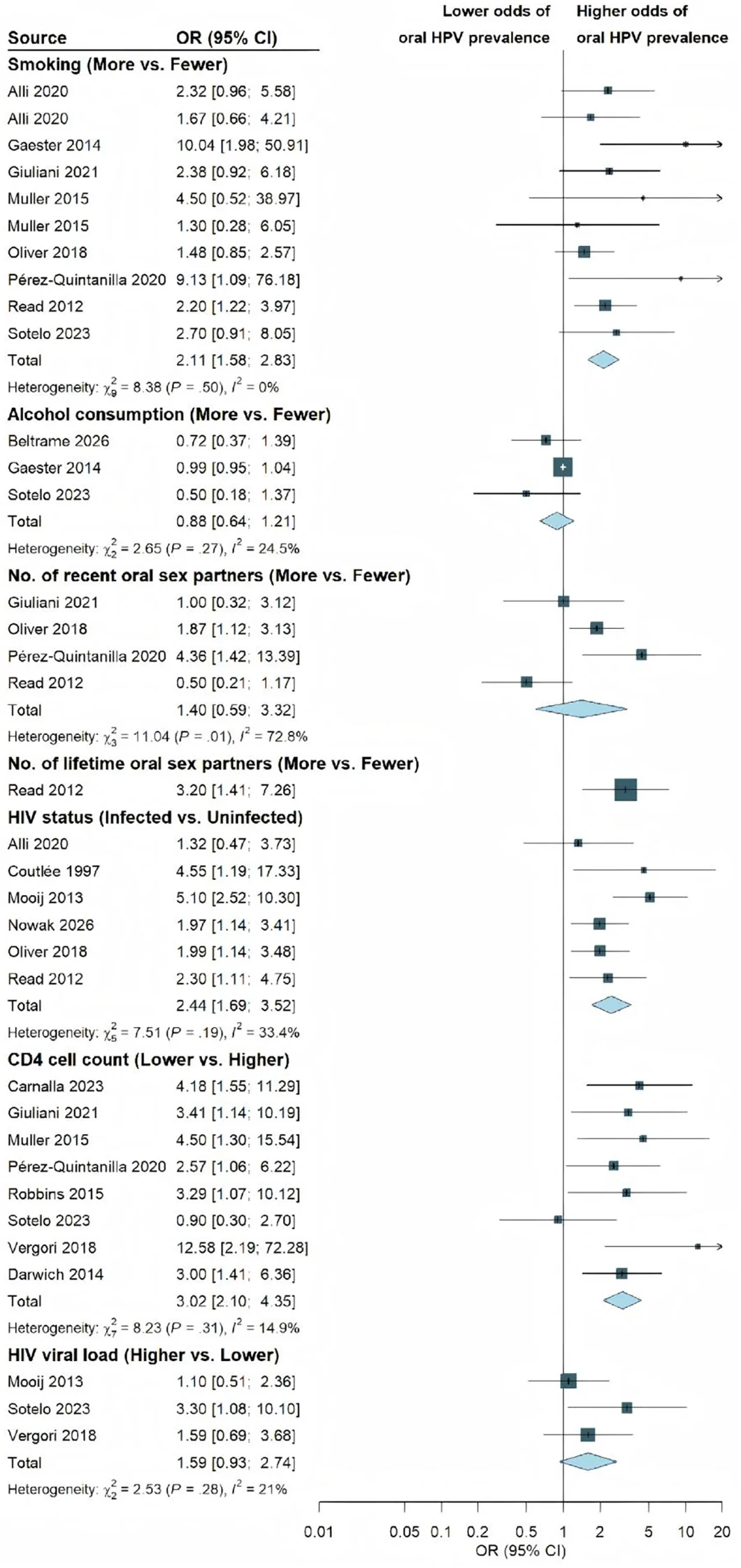
Forest plot of the factors associated with oral HPV prevalence. (+) denotes HIV-positive, (-) denotes HIV-negative.

Subgroup analyses revealed no statistically significant between-group differences according to HPV type, HIV status, or geographical region (all P for subgroup differences > 0.05) (**Supplementary Table S7**). The certainty of evidence was rated as low for HIV status, CD4 cell count, and lifetime oral-sex partners, and very low for the remaining factors (**Table 2**). The funnel plot and Egger’s and Begg’s tests suggested publication bias for the association between smoking and oral HPV prevalence (P = 0.032 and 0.025, respectively) (**Supplementary Figure S1**).

**Table 2:** Evaluation of quality of evidence for HPV prevalence using the grading of recommendations assessment, development and evaluation (GRADE) framework.

Outcomes	Pooled outcomes (95% CI)	Number of participants (number of included studies)	Statistical heterogeneity	Risk of bias	Imprecision	Inconsistency	Indirectness	Publication bias	Quality of evidence (GRADE)
Smoking (More vs. Fewer)	OR 2.11 (1.58-2.83)	2,713 (8 studies with 10 data points)	I²=0%; P = 0.497	Not serious	Not serious	Not serious	Not serious	Serious; Egger P = 0.032, Begg P = 0.025	⊕○○○ Very low
Alcohol consumption (More vs. Fewer)	OR 0.88 (0.64-1.21)	1,115 (3 studies with 3 data points)	I²=24.5%; P = 0.266	Not serious	Serious	Not serious	Not serious	Not assessable; Egger P = 0.098, Begg P = 0.117	⊕○○○ Very low
No. of recent oral sex partners (More vs. Fewer)	OR 1.40 (0.59-3.32)	1713 (4 studies with 4 data points)	I²=72.8%; P = 0.012	Not serious	Very serious	Serious	Not serious	Not assessable; Egger P = 0.436, Begg P = 0.602	⊕○○○ Very low
No. of lifetime oral sex partners (More vs. Fewer)	OR 3.20 (1.41-7.26)	500 (1 study with 1 data point)	NA	Not serious	Serious	Not assessable	Not serious	Not assessable	⊕⊕○○ Low
HIV status (Infected vs. Uninfected)	OR 2.44 (1.69-3.52)	3,355 (6 studies with 6 data points)	I²=33.4%; P = 0.185	Not serious	Not serious	Not serious	Not serious	Not assessable; Egger P = 0.651, Begg P = 0.573	⊕⊕○○ Low
CD4 cell count (Lower vs. Higher)	OR 3.02 (2.10-4.35)	2,224 (8 studies with 8 data points)	I²=14.9%; P = 0.313	Not serious	Not serious	Not serious	Not serious	Not assessable; Egger P = 0.330, Begg P = 0.216	⊕⊕○○ Low
HIV viral load (Higher vs. Lower)	OR 1.59 (0.93-2.74)	752 (3 studies with 3 data points)	I²=21.0%; P = 0.282	Not serious	Serious	Not serious	Not serious	Not assessable; Egger P = 0.098, Begg P = 0.117	⊕○○○ Very low

The quality of evidence for observational studies begins at “Low” when assessing a causal effect, whereas the quality for randomized controlled trials (RCTs) starts at “High.” The overall certainty of evidence can be downgraded based on five criteria: risk of bias, imprecision, inconsistency, indirectness, and publication bias. Conversely, the quality can be upgraded for the following reasons: a large effect size, evidence of a dose-response gradient, or if all plausible residual confounding would increase confidence in the estimated effect.

### 3.3 Factors associated with any oral HPV incidence

For oral HPV incidence, smoking was not significantly associated with the hazard of acquiring oral HPV infection across 5 studies (HR, 1.26; 95% CI, 0.83-1.92; I² = 54.0%) (**Figure 3**). Similarly, alcohol consumption showed no significant association with incident infection based on 2 estimates from a single study (HR, 0.90; 95% CI, 0.50-1.64; I² = 0%). In contrast, a greater number of recent oral-sex partners was significantly associated with increased oral HPV incidence across 3 studies (HR, 1.88; 95% CI, 1.41-2.49; I² = 0%). Greater lifetime numbers of oral-sex partners were also associated with an increased hazard of infection across 3 studies contributing 4 estimates (HR, 2.19; 95% CI, 1.03-4.68), although substantial heterogeneity was observed (I² = 68.4%). Regarding HIV-related factors, HIV infection was associated with a significantly increased hazard of incident oral HPV infection across 2 studies (HR, 1.90; 95% CI, 1.20-3.02; I² = 56.5%). Lower CD4 cell counts were similarly associated with increased incidence across 4 studies (HR, 1.85; 95% CI, 1.43-2.40; I² = 0%). Higher HIV viral load was also significantly associated with an increased hazard of incident oral HPV infection across 2 studies (HR, 2.51; 95% CI, 1.28-4.93; I² = 0.2%).

**Figure 3:**
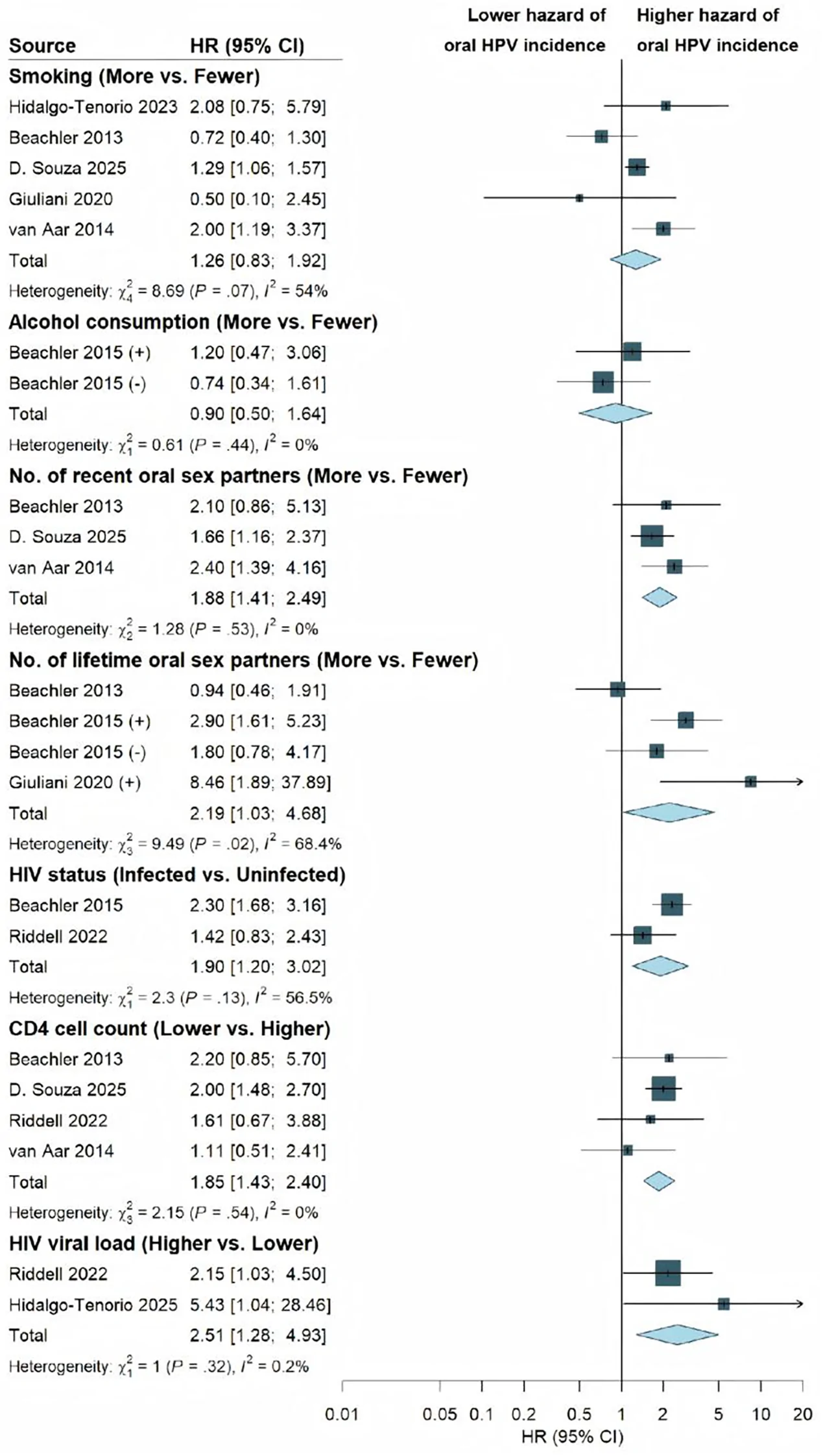
Forest plot of the factors associated with oral HPV incidence. (+) denotes HIV-positive, (-) denotes HIV-negative.

No statistically significant differences were identified between subgroups defined by HPV type, HIV status, or geographical region (all P for subgroup differences > 0.05) (**Supplementary Table S8**). The certainty of evidence was low for recent oral-sex partners and CD4 cell count and very low for the other investigated factors (**Table 3**).

**Table 3:** Evaluation of quality of evidence for HPV incidence Using the grading of recommendations assessment, development and evaluation (GRADE) framework.

Outcomes	Pooled outcomes (95% CI)	Number of participants (number of included studies)	Statistical heterogeneity	Risk of bias	Imprecision	Inconsistency	Indirectness	Publication bias	Quality of evidence (GRADE)
Smoking (More vs. Fewer)	HR 1.26 (0.83-1.92)	3,155 (5 studies with 5 data points)	I²=54.0%; P = 0.069	Not serious	Serious	Serious	Serious	Not assessable; Egger P = 0.825, Begg P = 0.624	⊕○○○ Very low
Alcohol consumption (More vs. Fewer)	HR 0.90 (0.50-1.64)	1,230 (1 study with 2 data points)	I²=0%; P = 0.436	Not serious	Serious	Not assessable	Not serious	Not assessable	⊕○○○ Very low
No. of recent oral sex partners (More vs. Fewer)	HR 1.88 (1.41-2.49)	2,714 (3 studies with 3 data points)	I²=0%; P = 0.526	Not serious	Not serious	Not serious	Not serious	Not assessable; Egger P = 0.519, Begg P = 0.602	⊕⊕○○ Low
No. of lifetime oral sex partners (More vs. Fewer)	HR 2.19 (1.03-4.68)	1,737 (3 studies with 4 data points)	I²=68.4%; P = 0.023	Not serious	Serious	Serious	Not serious	Not assessable; Egger P = 0.609, Begg P = 0.497	⊕○○○ Very low
HIV status (Infected vs. Uninfected)	HR 1.90 (1.20-3.02)	1,673 (2 studies with 2 data points)	I²=56.5%; P = 0.129	Not serious	Not serious	Serious	Not serious	Not assessable	⊕○○○ Very low
CD4 cell count (Lower vs. Higher)	HR 1.85 (1.43-2.40)	2,724 (4 studies with 4 data points)	I²=0%; P = 0.542	Not serious	Not serious	Not serious	Not serious	Not assessable; Egger P = 0.467, Begg P = 1.000	⊕⊕○○ Low
HIV viral load (Higher vs. Lower)	HR 2.51 (1.28-4.93)	521 (2 studies with 2 data points)	I²=0.2%; P = 0.317	Not serious	Serious	Not serious	Serious†	Not assessable	⊕○○○ Very low

The quality of evidence for observational studies begins at “Low” when assessing a causal effect, whereas the quality for randomized controlled trials (RCTs) starts at “High.” The overall certainty of evidence can be downgraded based on five criteria: risk of bias, imprecision, inconsistency, indirectness, and publication bias. Conversely, the quality can be upgraded for the following reasons: a large effect size, evidence of a dose-response gradient, or if all plausible residual confounding would increase confidence in the estimated effect.

### 3.4 Factors associated with oral HPV clearance

For oral HPV clearance, smoking was associated with a lower estimated clearance hazard across 2 studies contributing 3 effect estimates, although the association did not reach statistical significance (HR, 0.75; 95% CI, 0.56-1.01; I² = 0%) (**Figure 4**). The number of oral-sex partners was not significantly associated with HPV clearance across 2 studies (HR, 0.81; 95% CI, 0.05-14.37), with substantial between-study heterogeneity (I² = 92.2%). HIV infection was associated with a lower estimated hazard of oral HPV clearance across 3 studies (HR, 0.43; 95% CI, 0.19-1.00), although the confidence interval reached the null value and substantial heterogeneity was observed (I² = 88.3%). Similarly, lower CD4 cell counts were associated with a reduced estimated clearance hazard across 3 studies, but the association was not statistically significant (HR, 0.65; 95% CI, 0.27-1.53; I² = 60.5%). Higher HIV viral load was also associated with a lower estimated clearance hazard in the single contributing study, although the association did not reach statistical significance (HR, 0.15; 95% CI, 0.02-1.12). Overall, the associations between the investigated factors and oral HPV clearance remained inconclusive, with very-low-certainty evidence for all evaluated factors (**Table 4**).

**Figure 4:**
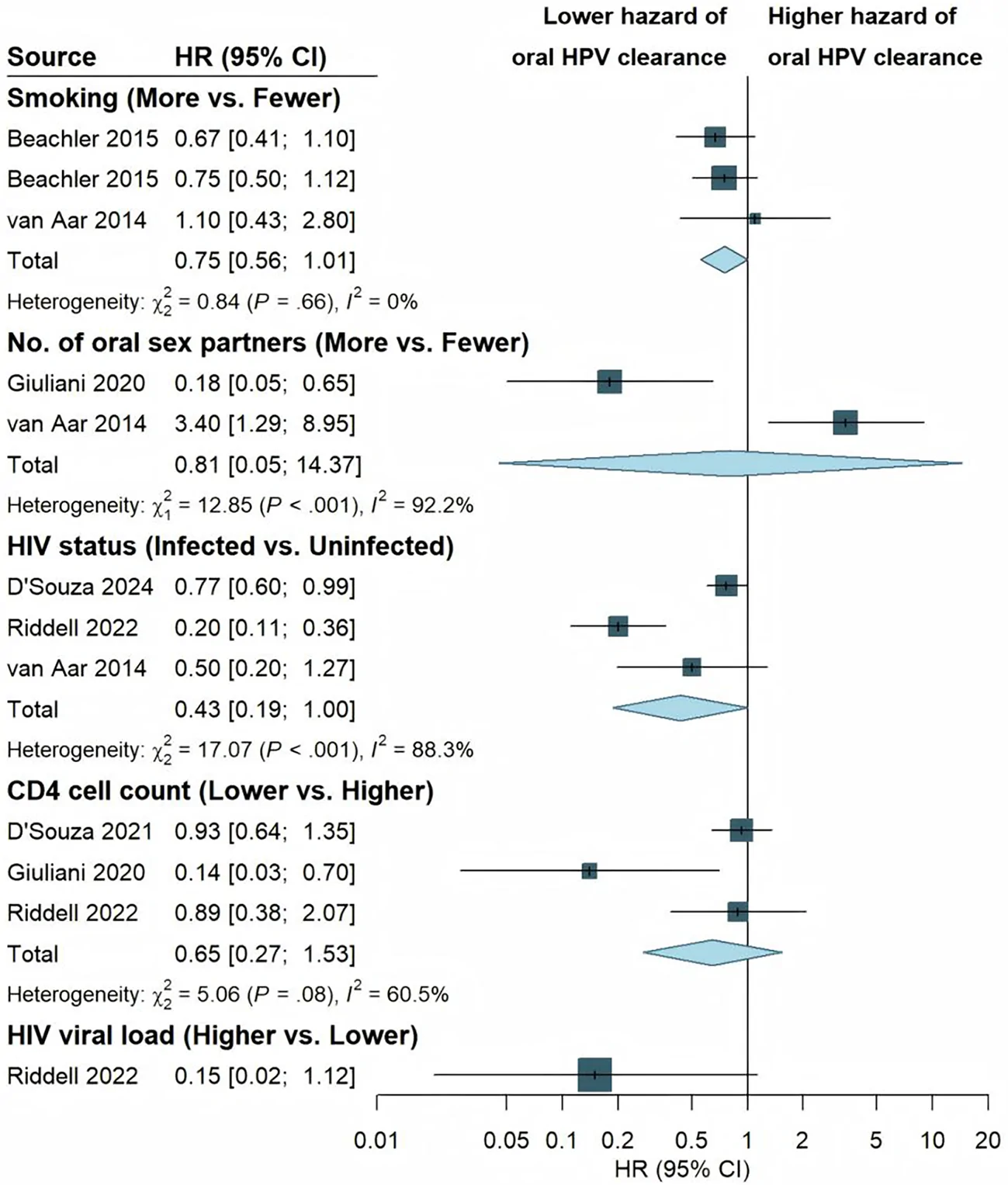
Forest plot of the factors associated with oral HPV clearance. (+) denotes HIV-positive, (-) denotes HIV-negative.

**Table 4:** Evaluation of quality of evidence for HPV clearance using the grading of recommendations assessment, development and evaluation (GRADE) framework.

Outcomes	Pooled outcomes (95% CI)	Number of participants (number of included studies)	Statistical heterogeneity	Risk of bias	Imprecision	Inconsistency	Indirectness	Publication bias	Quality of evidence (GRADE)
Smoking (More vs. Fewer)	HR 0.75 (0.56-1.01)	1,953 (2 studies with 3 data points)	I²=0%; P = 0.656	Not serious	Serious	Not serious	Not serious	Not assessable; Egger P = 0.419, Begg P = 0.602	⊕○○○ Very low
No. of oral sex partners (More vs. Fewer)	HR 0.81 (0.05-14.37)	864 (2 studies with 2 data points)	I²=92.2%; P<0.001	Not serious	Very serious	Very serious	Not serious	Not assessable	⊕○○○ Very low
HIV status (Infected vs. Uninfected)	HR 0.43 (0.19-1.00)	1,601 (3 studies with 3 data points)	I²=88.3%; P<0.001	Not serious	Serious	Serious	Not serious	Not assessable; Egger P = 0.495, Begg P = 0.602	⊕○○○ Very low
CD4 cell count (Lower vs. Higher)	HR 0.65 (0.27-1.53)	993 (3 studies with 3 data points)	I²=60.5%; P = 0.080	Not serious	Very serious	Serious	Not serious	Not assessable; Egger P = 0.372, Begg P = 0.117	⊕○○○ Very low
HIV viral load (Higher vs. Lower)	HR 0.15 (0.02-1.12)	443 (1 study with 1 data point)	NA	Not serious	Very serious	Not assessable	Not serious	Not assessable	⊕○○○ Very low

The quality of evidence for observational studies begins at “Low” when assessing a causal effect, whereas the quality for randomized controlled trials (RCTs) starts at “High.” The overall certainty of evidence can be downgraded based on five criteria: risk of bias, imprecision, inconsistency, indirectness, and publication bias. Conversely, the quality can be upgraded for the following reasons: a large effect size, evidence of a dose-response gradient, or if all plausible residual confounding would increase confidence in the estimated effect.

## 4 Discussion

This systematic review and meta-analysis demonstrate that behavioral and HIV-related factors are associated with oral HPV prevalence and incidence in distinct patterns. HIV infection and lower CD4 cell counts were consistently associated with both prevalent and incident oral HPV infection, highlighting the importance of HIV-related immunosuppression across different stages of infection. In contrast, smoking was significantly associated with prevalence but not incidence, whereas recent oral-sex exposure and higher HIV viral load showed clearer associations with incident infection. These findings underscore the importance of distinguishing prevalence from incidence when evaluating oral HPV determinants, as prevalent infection reflects both acquisition and infection duration. This distinction is clinically relevant because persistent oncogenic oral HPV infection is implicated in the development of HPV-associated oropharyngeal cancer (D'Souza et al., 2024; Lim and D'Silva, 2024; D'Souza et al., 2025).

The associations of HIV infection and lower CD4 cell counts with both oral HPV prevalence and incidence are consistent with previous evidence showing a greater burden of oral high-risk HPV among PLWH (Dube Mandishora et al., 2025). A multicenter cohort similarly reported higher oral HPV prevalence in HIV-positive adults and greater persistence among those with a history of poorly controlled HIV (Riddell et al., 2022). The associations with lower CD4 counts are biologically plausible because impaired cell-mediated immunity may compromise HPV-specific immune surveillance and facilitate both infection acquisition and prolonged viral detection (Beachler et al., 2015; Carnalla et al., 2023). Interestingly, higher HIV viral load was significantly associated with oral HPV incidence (HR, 2.51; 95% CI, 1.28-4.93), but not prevalence (OR, 1.59; 95% CI, 0.93-2.74). This finding suggests that HIV virologic activity may be particularly relevant to newly detected infections, although the limited number of contributing studies warrants cautious interpretation. The certainty of evidence for HIV-related factors ranged from low to very low, indicating the need for further longitudinal investigation.

Smoking, a potentially modifiable exposure, was significantly associated with oral HPV prevalence (OR, 2.11; 95% CI, 1.58-2.83), but not incidence (HR, 1.26; 95% CI, 0.83-1.92). This difference may indicate that smoking contributes to the burden of detectable oral HPV through mechanisms beyond initial acquisition, potentially including prolonged infection duration or impaired local mucosal immunity (Alli et al., 2020; D'Souza et al., 2025). However, the association between smoking and reduced HPV clearance did not reach statistical significance, and this interpretation remains speculative. In contrast, a greater number of recent oral-sex partners was significantly associated with oral HPV incidence (HR, 1.88; 95% CI, 1.41-2.49), but not prevalence (OR, 1.40; 95% CI, 0.59-3.32), supporting the relevance of recent sexual exposure to newly detected infection (Beachler et al., 2015; Dube Mandishora et al., 2024; D'Souza et al., 2025). Greater lifetime oral-sex exposure was associated with both outcomes, although the prevalence estimate was derived from a single study and substantial heterogeneity was observed in the incidence analysis. These findings suggest that behavioral factors may have different associations with prevalent and incident oral HPV infection, emphasizing the value of evaluating these outcomes separately.

Alcohol consumption was not significantly associated with either oral HPV prevalence (OR, 0.88; 95% CI, 0.64-1.21) or incidence (HR, 0.90; 95% CI, 0.50-1.64). However, the incidence estimate was derived from a single study, and the available evidence remains insufficient to exclude a potential association (Sotelo and Deluca, 2023; Beltrame et al., 2026b). Oral and anal HPV infections can coexist in high-risk men, but genotype concordance is often limited, indicating that detection at one mucosal site is an imperfect proxy for infection at another anatomical site (Rollo et al., 2021). The very high oral and cervical HPV prevalence reported in one cohort of women with HIV illustrates how population selection and laboratory methods can produce extreme estimates, underscoring the need for standardized exposure and outcome definitions (Pérez-Quintanilla et al., 2020).

Evidence regarding oral HPV clearance was less conclusive than that for prevalence and incidence. None of the investigated behavioral or HIV-related factors showed a statistically significant association with clearance. HIV infection was associated with a lower estimated clearance hazard (HR, 0.43; 95% CI, 0.19-1.00), although substantial heterogeneity was observed and the certainty of evidence was very low. These findings are broadly consistent with previous longitudinal studies suggesting that HIV-related immunosuppression may contribute to prolonged oral HPV detection (Riddell et al., 2022; D'Souza et al., 2024; Dickey et al., 2025). Differences in follow-up duration, HPV genotype definitions, and the number of consecutive negative tests required to confirm clearance may also contribute to inconsistent findings across studies (Wong et al., 2020; D'Souza et al., 2024). Importantly, studies reporting HPV persistence rather than clearance were included in the systematic review but not quantitatively pooled with clearance outcomes. Further prospective studies using standardized definitions and repeated HPV measurements are needed to clarify the factors influencing infection clearance.

Several limitations should be acknowledged. All included studies were observational, and residual confounding and reverse causation cannot be excluded. Furthermore, incidence and clearance analyses were based on relatively few studies, and differences in exposure definitions, HPV genotypes, and follow-up procedures may have contributed to heterogeneity. Evidence regarding oral HPV clearance and alcohol consumption was particularly limited. In addition, Egger’s and Begg’s tests were applied to some analyses involving only two to five studies, for which these tests have very low statistical power. Therefore, the results of publication bias assessments should be interpreted with caution.

## 5 Conclusion

Oral HPV prevalence and incidence in PLWH and populations at increased risk of HIV acquisition are associated with distinct behavioral and immunologic factors. HIV infection and lower CD4 cell counts were associated with both prevalent and incident oral HPV infection, whereas smoking showed a clearer association with prevalence and recent oral-sex exposure and higher HIV viral load with incidence. Evidence regarding HPV clearance remained inconclusive. These findings highlight the importance of distinguishing infection prevalence from incidence when evaluating oral HPV determinants and may inform future longitudinal research and targeted prevention strategies.

## Data Availability

The original contributions presented in the study are included in the article/**Supplementary Material**. Further inquiries can be directed to the corresponding authors.
