# Supplementary material for: Factors associated with oral HPV infection and clearance in people living with HIV and populations at increased risk of HIV acquisition: a systematic review and meta-analysis
Source: Front Cell Infect Microbiol. 2026 Oct 29;16:1963308. doi: 10.3389/fcimb.2026.1963308 (PMC13754742; doi:10.3389/fcimb.2026.1963308)
Supplement: Supplementary file 1 [file Table1.docx]

Table S1: Search Strategy

| Database | Search Term | n |
| --- | --- | --- |
| Embase | ('human immunodeficiency virus infection'/exp OR 'human immunodeficiency virus'/exp OR hiv:ti,ab,kw OR 'hiv 1':ti,ab,kw OR 'hiv 2':ti,ab,kw OR aids:ti,ab,kw OR 'acquired immunodeficiency syndrome':ti,ab,kw OR 'HIV positive':ti,ab,kw OR 'HIV-positive':ti,ab,kw OR 'HIV infected':ti,ab,kw OR 'HIV-infected':ti,ab,kw OR 'people living with HIV':ti,ab,kw OR plwh:ti,ab,kw OR plhiv:ti,ab,kw) AND ('papillomavirus infection'/exp OR 'human papillomavirus'/exp OR 'human papillomavirus 16'/exp OR hpv:ti,ab,kw OR hpvs:ti,ab,kw OR 'human papillomavirus':ti,ab,kw OR 'human papilloma virus':ti,ab,kw OR papillomavirus*:ti,ab,kw) AND ('mouth'/exp OR 'mouth mucosa'/exp OR 'oral cavity'/exp OR 'oropharynx'/exp OR 'saliva'/exp OR oral:ti,ab,kw OR mouth:ti,ab,kw OR 'oral cavity':ti,ab,kw OR 'oral mucosa':ti,ab,kw OR oropharyn*:ti,ab,kw OR tonsil*:ti,ab,kw OR saliva*:ti,ab,kw OR salivary:ti,ab,kw OR 'oral rinse':ti,ab,kw OR 'oral rinses':ti,ab,kw OR gargle*:ti,ab,kw) AND ('incidence'/exp OR 'risk factor'/exp OR incidence:ti,ab,kw OR incident:ti,ab,kw OR acquisition:ti,ab,kw OR acquired:ti,ab,kw OR clearance:ti,ab,kw OR cleared:ti,ab,kw OR clear*:ti,ab,kw OR persistence:ti,ab,kw OR persistent:ti,ab,kw OR 'natural history':ti,ab,kw OR predictor*:ti,ab,kw OR determinant*:ti,ab,kw OR 'risk factor*':ti,ab,kw) AND [humans]/lim | 844 |
| PubMed | ("HIV Infections"[Mesh] OR HIV[tiab] OR HIV-1[tiab] OR HIV-2[tiab] OR AIDS[tiab] OR "acquired immunodeficiency syndrome"[tiab] OR "HIV positive"[tiab] OR HIV-positive[tiab] OR "HIV infected"[tiab] OR HIV-infected[tiab] OR "people living with HIV"[tiab] OR PLWH[tiab] OR PLHIV[tiab]) AND ("Papillomavirus Infections"[Mesh] OR "Papillomaviridae"[Mesh] OR "Human papillomavirus 16"[Mesh] OR HPV[tiab] OR HPVs[tiab] OR "human papillomavirus"[tiab] OR "human papilloma virus"[tiab] OR papillomavirus*[tiab]) AND ("Mouth"[Mesh] OR "Mouth Mucosa"[Mesh] OR "Oropharynx"[Mesh] OR "Saliva"[Mesh] OR oral[tiab] OR mouth[tiab] OR "oral cavity"[tiab] OR "oral mucosa"[tiab] OR oropharyn*[tiab] OR tonsil*[tiab] OR saliva*[tiab] OR salivary[tiab] OR "oral rinse"[tiab] OR "oral rinses"[tiab] OR gargle*[tiab]) AND (incidence[tiab] OR incident[tiab] OR acquisition[tiab] OR acquired[tiab] OR clearance[tiab] OR cleared[tiab] OR clear*[tiab] OR persistence[tiab] OR persistent[tiab] OR "natural history"[tiab] OR "risk factor*"[tiab] OR predictor*[tiab] OR determinant*[tiab] OR "Incidence"[Mesh] OR "Risk Factors"[Mesh]) NOT (animals[mh] NOT humans[mh]) | 408 |
| Cochrane Library | (HIV OR "HIV-1" OR "HIV 1" OR "HIV-2" OR "HIV 2" OR AIDS OR "acquired immunodeficiency syndrome" OR "HIV positive" OR "HIV-positive" OR "HIV infected" OR "HIV-infected" OR "people living with HIV" OR PLWH OR PLHIV) AND (HPV OR HPVs OR "human papillomavirus" OR "human papilloma virus" OR papillomavirus*) AND (oral OR mouth OR "oral cavity" OR "oral mucosa" OR oropharyn* OR tonsil* OR saliva* OR salivary OR "oral rinse" OR "oral rinses" OR gargle*) AND (inciden* OR prevalen* OR acquisition OR acquired OR clearance OR clearances OR cleared OR clearing OR persistence OR persistent OR "natural history" OR predictor* OR determinant* OR association* OR associated OR correlat* OR (risk NEAR/1 factor*)) | 88 |

Table S2. Risk of Bias Assessment of Cross-Sectional Studies using Newcastle-Ottawa Scale

| **First Author, Year** | **Representativeness of Sample** | **Non-Respondents** | **Sample Size Justified and Satisfactory** | **Ascertainment of the Exposure** | **Adjusted for Age** | **Adjusted for other factors** | **Assessment of Outcomes by Record Linkage** | **Assessment of Outcome and Statistical Check** | **Total Score /9** | **Risk of Bias** |
| --- | --- | --- | --- | --- | --- | --- | --- | --- | --- | --- |
| Alli et al., 2020 | 1 | 0 | 0 | 1 | 1 | 1 | 1 | 1 | **6** | **Moderate** |
| Beltrame et al., 2026 | 0 | 1 | 0 | 1 | 1 | 1 | 1 | 1 | **6** | **Moderate** |
| Carnalla et al., 2023 | 0 | 0 | 0 | 1 | 1 | 1 | 1 | 1 | **5** | **Moderate** |
| Coutlée et al., 1997 | 1 | 1 | 0 | 1 | 0 | 1 | 1 | 1 | **6** | **Moderate** |
| Gaester et al., 2014 | 0 | 0 | 0 | 1 | 0 | 1 | 1 | 1 | **4** | **Moderate** |
| Giuliani et al., 2021 | 0 | 0 | 0 | 1 | 1 | 1 | 1 | 1 | **5** | **Moderate** |
| Mooij et al., 2013 | 1 | 1 | 0 | 2 | 1 | 1 | 1 | 1 | **8** | **Low** |
| Muller et al., 2015 | 1 | 0 | 0 | 1 | 1 | 1 | 1 | 1 | **6** | **Moderate** |
| Nowak et al., 2026 | 1 | 1 | 0 | 2 | 0 | 1 | 1 | 1 | **7** | **Low** |
| Oliver et al., 2018 | 0 | 0 | 0 | 1 | 1 | 1 | 1 | 1 | **5** | **Moderate** |
| Pérez-Quintanilla et al., 2020 | 0 | 0 | 0 | 1 | 0 | 0 | 1 | 1 | **3** | **High** |
| Read et al., 2012 | 1 | 1 | 1 | 1 | 1 | 1 | 1 | 1 | **8** | **Low** |
| Robbins et al., 2015 | 0 | 1 | 0 | 1 | 1 | 1 | 1 | 1 | **6** | **Moderate** |
| Sotelo and Deluca, 2023 | 0 | 0 | 0 | 1 | 0 | 0 | 1 | 1 | **3** | **High** |
| Vergori et al., 2018 | 1 | 0 | 0 | 1 | 1 | 1 | 1 | 1 | **6** | **Moderate** |
| 7-9 Stars: Low Risk of Bias, 4-6 Stars: Moderate Risk of Bias, 0-3 Stars: High Risk of Bias | | | | | | | | | | |

Table S3. Risk of Bias Assessment of Cohort Studies using Newcastle-Ottawa Scale

| **First Author, Year** | **Representative of Exposed Cohort** | **Selection of Non-Exposed Cohort** | **Ascertainment of Exposure** | **Demonstration that Outcome of Interest was Not Present at Start of Study** | **Adjusted for Age** | **Adjusted for other factors** | **Assessment of Outcomes by Record Linkage** | **Minimum 30-Day Follow-Up** | **Complete Follow-Up** | **Total Score /9** | **Risk of Bias** |
| --- | --- | --- | --- | --- | --- | --- | --- | --- | --- | --- | --- |
| Beachler et al., 2013 | 0 | 1 | 1 | 1 | 1 | 1 | 1 | 1 | 0 | **7** | **Low** |
| Beachler et al., 2015 | 1 | 1 | 1 | 1 | 1 | 1 | 1 | 1 | 0 | **8** | **Low** |
| D’Souza et al., 2025 | 1 | 1 | 1 | 1 | 1 | 1 | 1 | 1 | 1 | **9** | **Low** |
| D’Souza et al., 2007 | 0 | 1 | 1 | 1 | 1 | 1 | 1 | 1 | 1 | **8** | **Low** |
| D’Souza et al., 2021 | 0 | 1 | 1 | 1 | 1 | 1 | 1 | 1 | 0 | **7** | **Low** |
| D’Souza et al., 2024 | 1 | 1 | 1 | 1 | 1 | 1 | 1 | 1 | 1 | **9** | **Low** |
| Darwich et al., 2014 | 1 | 1 | 1 | 1 | 1 | 1 | 1 | 1 | 1 | **9** | **Low** |
| Giuliani et al., 2020 | 0 | 1 | 1 | 1 | 1 | 1 | 1 | 1 | 0 | **7** | **Low** |
| Hidalgo-Tenorio et al., 2025 | 1 | 1 | 1 | 1 | 1 | 1 | 1 | 1 | 1 | **9** | **Low** |
| Mooij et al., 2014 | 1 | 1 | 1 | 1 | 1 | 1 | 1 | 1 | 1 | **9** | **Low** |
| Riddell et al., 2022 | 0 | 0 | 1 | 1 | 0 | 1 | 1 | 1 | 0 | **5** | **Moderate** |
| van Aar et al., 2014 | 1 | 1 | 1 | 1 | 1 | 1 | 1 | 1 | 1 | **9** | **Low** |
| 7-9 Stars: Low Risk of Bias, 4-6 Stars: Moderate Risk of Bias, 0-3 Stars: High Risk of Bias | | | | | | | | | | | |

Table S4. Original effect sizes and comparisons for the outcome of HPV prevalence.

| Author | HIV Status | HPV Type | Effect Measure | Smoking | Alcohol Use | Recent Oral-Sex Partners | Lifetime Oral-Sex Partners | HIV Infection | CD4 | HIV Viral Load | Model Adjustments |
| --- | --- | --- | --- | --- | --- | --- | --- | --- | --- | --- | --- |
| Alli 2020 | HIV-positive | HR-HPV | aOR | Ever smoker (15.4 pack-years) vs never: 3.46 (1.01–11.94) | — | — | — | — | — | — | Adjusted for age, sex, race/ethnicity, lifetime sex partners, oral-sex history, oral lesions, and pack-years; included HIV × smoking interaction. |
|  | HIV-negative | HR-HPV | aOR | Ever smoker (15.4 pack-years) vs never: 1.56 (0.39–7.86) | — | — | — | — | — | — |  |
|  | HIV-positive | Any α-HPV | aOR | Ever smoker (15.4 pack-years) vs never: 2.32 (0.96–5.56) | — | — | — | — | — | — |  |
|  | HIV-negative | Any α-HPV | aOR | Ever smoker (15.4 pack-years) vs never: 1.67 (0.68–4.32) | — | — | — | — | — | — |  |
|  | Mixed | Any α-HPV | aOR | — | — | — | — | HIV-positive vs HIV-negative: 1.32 (0.47–3.76) | — | — |  |
|  | Mixed | HR-HPV | aOR | — | — | — | — | HIV-positive vs HIV-negative: 1.59 (0.32–8.73) | — | — |  |
| Beltrame 2026 | HIV-positive | HR-HPV | aOR | — | 3–15 vs 0–2 drinks/mo: 0.91 (0.52–1.60); ≥16 vs 0–2 drinks/mo: 0.72 (0.37–1.38) | — | — | — | — | — | Adjusted for age, country, lifetime female sex partners, and smoking status. |
|  | HIV-positive | LR-HPV | aOR | — | 3–15 vs 0–2 drinks/mo: 1.11 (0.68–1.84); ≥16 vs 0–2 drinks/mo: 0.95 (0.53–1.68) | — | — | — | — | — |  |
| Coutlée 1997 | Mixed | Any HPV | aOR | — | — | — | — | HIV-positive vs HIV-negative: 4.55 (1.19–17.26) | — | — | Variables assessed individually: sex, sexual orientation, number of sex partners, HIV status, age, prior STDs, and unprotected oral sex. |
| Carnalla 2023 | Mixed | HR-HPV | aOR | — | — | — | — | — | CD4 200–499 vs CD4 ≥500 cells/μL: aOR 4.18 (95% CI 1.55–11.30) | — | Adjusted for age, joint HIV/CD4 category, and recent recreational vasodilator use. |
| Gaester 2014 | HIV-positive | Any HPV | aOR | Smoking variable coding not explicitly defined: 10.04 (1.98–50.92) | Drinker vs non-drinker: 0.99 (0.94–1.03) | — | — | — | — | — | Forward stepwise multivariable logistic regression; candidates selected from bivariable analyses. |
| Giuliani 2021 | HIV-positive | Any α-HPV | aOR | Current vs non-current: 2.38 (0.92–6.21) | — | Receptive oral-sex partners in past 6 mo, >3 vs ≤3: 1.00 (0.32–3.12) | — | — | — | — | Stratified by HIV status and HPV genus; age forced in; univariable P<0.10 factors added; oral-sex variables prioritized among correlated behaviors. |
|  | HIV-positive | LR-HPV | aOR | — | — | Receptive oral-sex partners in past 6 mo, >3 vs ≤3: 0.75 (0.27–2.03) | — | — | Current CD4 <500 vs ≥500 cells/μL: 3.41 (1.14–10.18) | — |  |
| Mooij 2013 | Mixed | Any HPV | aOR | — | — | — | — | HIV-positive vs HIV-negative: 5.1 (2.5–10.2) | — | — | Adjusted for age, HIV status, and behavioral factors. |
|  | HIV-negative | HR-HPV | aOR | Former vs never: 0.8 (0.3–2.0); Current vs never: 0.9 (0.4–2.1) | — | Past 6 mo, 3–8 vs ≤2: 1.1 (0.4–2.9); ≥9 vs ≤2: 1.7 (0.7–4.6) | — | — | — | — | Adjusted for age, smoking, lifetime male sex partners, past-6-mo oral-sex partners, and past-6-mo rimming. |
|  | HIV-positive | HR-HPV | aOR | — | — | — | — | — | — | ≥50 vs <50 copies/mL: 1.1 (0.5–2.3) | Adjusted for age, smoking, lifetime male sex partners, past-6-mo oral-sex partners, past-6-mo rimming, CD4, and HIV RNA. |
| Muller 2015 | HIV-negative | Any HPV | aOR | Former vs never: 3.3 (0.5–21.5); Current vs never: 4.5 (0.5–37.5) | — | — | — | — | — | — | Final model forced age and sex; also adjusted for lifetime sex partners, smoking, and cannabis use. |
|  | HIV-positive | Any HPV | aOR | Former vs never: 1.4 (0.4–5.2); Current vs never: 1.3 (0.3–6.5) | — | — | — | — | CD4 201–499 vs ≥500: 1.3 (0.5–3.5); ≤200 vs ≥500 cells/μL: 4.5 (1.3–15.5) | — | Final model forced age and sex; also adjusted for lifetime sex partners, sexual orientation, CD4, smoking, and cannabis use. |
| Nowak 2026 | Mixed | Any HPV | aOR | — | — | — | — | HIV-positive vs HIV-negative: 1.97 (1.14–3.41) | — | — | Adjusted for HIV status, years since anal sexual debut, **sexual position with male partners, ever had sex with a woman.** |
|  | Mixed | HR-HPV | aOR | — | — | — | — | HIV-positive vs HIV-negative: 2.30 (0.93–5.66) | — | — |  |
| Oliver 2018 | Mixed | Any HPV | aPR | Past-year cigarettes, ≤12 vs 0: 1.07 (0.57–2.00); >12 vs 0: 1.48 (0.85–2.57) | — | Partners performing oral sex on participant in past 3 mo, >5 vs ≤5: 1.87 (1.12–3.13) | — | Self-reported HIV-positive vs not HIV-positive: 1.99 (1.14–3.48) | — | — | Each model adjusted for age, race, smoking, and HIV status, except when the variable was the exposure. |
| Pérez-Quintanilla 2020 | HIV-positive | HPV16 | aOR | — | — | Oral-sex partners in past 6 mo, 2 vs 1: 4.58 (1.67–12.57); ≥3 vs 1: 8.04 (2.49–25.95) | — | — | ≤200 vs >500 cells/μL: 2.73 (1.06–7.00) | — | Adjusted for sociodemographic, clinical and sexual-behavior factors |
|  | HIV-positive | HPV16/18 | aOR | — | — | Oral-sex partners in past 6 mo, 2 vs 1: 3.41 (1.25–9.27); ≥3 vs 1: 4.36 (1.42–13.40) | — | — | ≤200 vs >500 cells/μL: 2.57 (1.04–6.09) | — |  |
|  | HIV-positive | HPV66 | aOR | — | — | Oral-sex partners in past 6 mo, 2 vs 1: 3.20 (1.16–8.79); ≥3 vs 1: 8.45 (2.48–28.70) | — | — | — | — |  |
|  | HIV-positive | HR-HPV | aOR | Smoker vs non-smoker: 9.13 (1.09–75.89) | — | — | — | — | — | — |  |
| Read 2012 (Model 1) | Mixed | Any HPV | aOR | Current vs non-current: 2.2 (1.2–3.9) | — | — | — | HIV-positive vs HIV-negative: 2.3 (1.1–4.7) | — | — | Adjusted for age, HIV status, current smoking, time since toothbrushing, history of anogenital warts, proportion of oral sex with ejaculation in the mouth, and lifetime deep-kissing partners. |
| Read 2012 (Model 2) | Mixed | Any HPV | aOR | Current vs non-current: 2.1 (1.2–3.7) | — | Penile oral-sex partners in past 2 wk: 1 vs 0: 1.4 (0.7–2.7); >1 vs 0: 0.5 (0.2–1.1); penile oral-sex partners from >2 wk to 1 yr ago: 2–9 vs 0–1: 0.5 (0.2–1.1); ≥10 vs 0–1: 1.5 (0.7–3.2) | Lifetime penile oral-sex partners, 26–100 vs 0–25: 2.8 (1.2–6.3); >100 vs 0–25: 3.2 (1.4–7.2) | HIV-positive vs HIV-negative: 2.1 (1.0–4.2) | — | — | Adjusted for age, HIV status, current smoking, time since toothbrushing, history of anogenital warts, proportion of oral sex with ejaculation in the mouth, and lifetime penile oral-sex partners. |
| Robbins 2015 | Mixed | Any HPV | aOR | — | — | — | — | — | CD4 <350 vs CD4 ≥350 cells/μL: aOR 3.29 (95% CI 1.07–10.13) | — | Final model adjusted for age, race/ethnicity, joint HIV/CD4 category, sex/sexual orientation, and recent oral sex performed on women. |
| Sotelo 2023 | HIV-positive | Any HPV | aOR | Smoker vs non-smoker: 2.70 (0.90–8.00) | Drinker vs non-drinker: 0.50 (0.20–1.50) | — | — | — | CD4% <29% vs ≥29%: 0.90 (0.30–2.70) | >40 vs ≤40 copies/mL: 3.30 (1.10–10.30) | Binary logistic model included age, sex/gender identity, sexual orientation, age at sexual debut, past-year sex partners, contraception, condom use for oral sex, oral sex, HPV vaccination, genital HPV history, smoking, alcohol, HIV RNA, and CD4%. |
|  | HIV-positive | HR-HPV | aOR | Smoker vs non-smoker: 1.60 (0.40–6.50) | Drinker vs non-drinker: 0.70 (0.20–2.90) | — | — | — | CD4% <29% vs ≥29%: 1.30 (0.30–4.90) | >40 vs ≤40 copies/mL: 3.90 (1.00–15.20) |  |
| Vergori 2018 | HIV-positive | Any HPV | aOR | — | — | — | — | — | <200 vs ≥200 cells/μL: 12.58 (2.19–72.30) | >40 vs ≤40/undetectable copies/mL: 1.59 (0.69–3.70) | Adjusted for age, past-12-mo lifetime sex partners, recreational drugs, CD4, HIV RNA, anal HPV, and wart history. |
| Darwich 2014 | HIV-positive | HPV16/18 | aOR | — | — | — | — | — | Nadir CD4 <200 vs ≥200 cells/mm³: 3.00 (1.40–6.30) | — | Adjusted for sexual-behavior group, age, duration of HIV infection, nadir and baseline CD4, HAART use and duration, smoking, alcohol, injection drug use, and sex partners. |

Table S5. Original effect sizes and comparisons for the outcome of HPV incidence.

| Author | HIV Status | HPV Type | Effect Measure | Smoking | Alcohol Use | Recent Oral-Sex Partners | Lifetime Oral-Sex Partners | HIV Infection | CD4 | HIV Viral Load | Model Adjustments |
| --- | --- | --- | --- | --- | --- | --- | --- | --- | --- | --- | --- |
| Beachler 2013 | HIV-positive | Any HPV | aHR | Current vs non-current: 0.72 (0.40–1.30) | — | Oral-sex partners in past 6 mo: 1 vs 0: 1.50 (0.70–3.20); ≥2 vs 0: 2.10 (0.87–5.20) | Lifetime oral-sex partners: 1–5 vs 0: 0.48 (0.22–1.00); ≥6 vs 0: 0.94 (0.46–1.90) | — | Nadir CD4: 100–250 vs >250: 1.70 (0.80–3.40); <100 vs >250: 2.20 (0.85–5.70); Current CD4: 200–499 vs ≥500: 0.69 (0.33–1.50); <200 vs ≥500: 0.49 (0.19–1.30) | — | Adjusted for age, sexual orientation, current smoking, lifetime and past-6-mo oral-sex partners, rimming history, nadir CD4, and current CD4. |
| Beachler 2015 | HIV-positive | Any HPV | aHR | Former vs never: 0.75 (0.39–1.40); Current vs never: 0.86 (0.42–1.80) | <2 vs 0 drinks/day: 0.98 (0.78–1.20); ≥2 vs 0 drinks/day: 1.20 (0.46–3.00) | Oral-sex partners in past 6 mo: 1 vs 0: 0.84 (0.66–1.10); 2–5 vs 0: 1.20 (0.86–1.70); ≥6 vs 0: 1.10 (0.62–1.90) | Lifetime oral-sex partners: 5–99 vs 0–4: 2.20 (1.20–4.00); ≥100 vs 0–4: 2.90 (1.60–5.20) | — | 200–499 vs >500 cells/μL: 1.40 (1.10–1.70); <200 vs >500 cells/μL: 3.50 (1.70–7.30) | — | Adjusted for HIV status, current CD4, age, sex, smoking, alcohol, site, tonsillectomy, recent toothbrushing, lifetime and recent oral-sex partners, and recent oral sex performed on women; all except tonsillectomy and lifetime oral-sex partners were time-updated. |
|  | HIV-negative | Any HPV | aHR | Former vs never: 1.10 (0.64–1.70); Current vs never: 1.00 (0.56–1.80) | <2 vs 0 drinks/day: 0.94 (0.61–1.40); ≥2 vs 0 drinks/day: 0.74 (0.34–1.60) | Oral-sex partners in past 6 mo: 1 vs 0: 0.84 (0.60–1.20); 2–5 vs 0: 1.40 (0.93–2.10); ≥6 vs 0: 2.30 (1.10–4.80) | Lifetime oral-sex partners: 5–99 vs 0–4: 1.30 (0.70–2.50); ≥100 vs 0–4: 1.80 (0.80–4.30) | — | — | — |  |
|  | Mixed | Any HPV | aHR | — | — | — | — | HIV-positive vs HIV-negative: 2.30 (1.70–3.20) | — | — | Adjusted for demographic, behavioral, and immunologic factors. |
| D. Souza 2025 | Mixed | Any HPV | aHR | Current vs non-current: 1.29 (1.05–1.56) | — | Oral-sex partners in past 6 mo: 2–5 vs 0–1: 1.54 (1.20–1.95); ≥6 vs 0–1: 1.66 (1.16–2.37) | — | — | CD4 200–500 vs CD4 >500 cells/μL: aOR 1.22 (95% CI 0.92–1.62); CD4 <200 vs CD4 >500 cells/μL: aOR 2.00 (95% CI 1.48–2.70) | — | Adjusted for age, current smoking, recent oral-sex partners, joint HIV/CD4 category, and other baseline oral HPV infection. |
| Giuliani 2020 | HIV-positive | Any HPV | aHR | — | — | — | Lifetime oral-sex partners, 25–95 vs <25: 1.58 (0.26–9.55); >95 vs <25: 8.46 (1.89–37.92) | — | — | — | Adjusted for age, current smoker, occasional partners for any sex, No. lifetime oral sex partners, No. recent oral sex partners and STI history. |
|  | HIV-negative | Any HPV | aHR | 0.25–18 vs 0 pack-years: 0.21 (0.05–0.93); >18 vs 0: 0.50 (0.10–2.41) | — | — | — | — | — | — |  |
| Riddell 2022 | Mixed | Any HPV | aHR | — | — | — | — | HIV-positive vs HIV-negative: 1.42 (0.83–2.43) | CD4 <200 vs ≥500: 1.61 (0.67–3.89); 200–499 vs ≥500: 1.22 (0.56–2.65) | ≥200 vs <200 copies/mL: 2.15 (1.03–4.51) | Adjusted for sex. |
| Hidalgo-Tenorio 2025 | HIV-positive | Any HPV | aHR | Smoker vs non-smoker: 2.082 (0.748–5.795) | — | — | — | — | — | HIV RNA <50 vs ≥50 copies/mL: 0.184 (0.035–0.959) | Adjusted for age, sex, past-12-mo sex partners, smoking, ART, duration of HIV infection and ART, current CD4, CD4/CD8 ratio, and HPV vaccination. |
| van Aar 2014 | Mixed | HR-HPV | aHR | Smoking only in past 6 mo vs neither: 2.00 (1.20–3.40); Smoking plus cannabis vs neither: 1.80 (1.00–3.00) | — | Oral-sex partners in past 6 mo: 3–7 vs ≤2: 1.00 (0.50–1.90); ≥8 vs ≤2: 2.40 (1.40–4.20) | — | HIV-positive vs HIV-negative: 3.80 (2.30–6.20) | — | — | Adjusted for HIV status, age, joint smoking/cannabis exposure, rimming, and past-6-mo oral-sex partners. |
|  | HIV-positive | HR-HPV | aHR | Smoking only in past 6 mo vs neither: 2.70 (1.40–5.20); Smoking plus cannabis: 2.00 (1.00–3.80) | — | Oral-sex partners in past 6 mo: 3–7 vs ≤2: 0.80 (0.40–1.70); ≥8 vs ≤2: 2.20 (1.10–4.40) | — | — | Nadir CD4 200–350 vs ≤199: 0.60 (0.30–1.00); ≥350 vs ≤199: 0.90 (0.40–1.90) | — | Adjusted for age, joint smoking/cannabis exposure, rimming, past-6-mo oral-sex partners, and nadir CD4. |

Table S6. Original effect sizes and comparisons for the outcome of HPV clearance or persistence.

| Study | HIV Status | HPV Type/Outcome | Effect Measure | Smoking | No. of Oral Sex Partners | HIV Infection | CD4 | HIV Viral Load | Model Adjustment |
| --- | --- | --- | --- | --- | --- | --- | --- | --- | --- |
| Beachler 2013 | HIV-positive | Any type-specific HPV (persistence) | aHR | Current vs non-current: 1.00 (0.67–1.60) | — | — | Nadir CD4: 100–250 vs >250: 0.87 (0.55–1.40); <100 vs >250: 1.40 (0.81–2.60); current CD4: 200–499 vs ≥500: 1.20 (0.70–2.00); ≤200 vs ≥500: 1.20 (0.59–2.30) | — | Adjusted for age, sexual orientation, prevalent vs incident infection, current smoking, nadir CD4, and current CD4. |
| Beachler 2015 | HIV-positive | Any HPV (clearance; 1 negative test) | aHR | Former vs never: 0.72 (0.48–1.10); current vs never: 0.67 (0.41–1.10) | — | — | 200–499 vs >500 cells/μL: 0.98 (0.83–1.20); <200 vs >500 cells/μL: 1.10 (0.74–1.70) | — | Wei–Lin–Weissfeld model; adjusted for current CD4, age, sex, infection type, smoking, and tonsillectomy; all variables except tonsillectomy were time-updated. |
|  | HIV-negative | Any HPV (clearance; 1 negative test) | aHR | Former vs never: 0.78 (0.54–1.10); current vs never: 0.75 (0.49–1.10) | — | — | — | — |  |
|  | Mixed | Any HPV (clearance; 2 consecutive negative tests) | aHR | — | — | HIV-positive vs HIV-negative: 0.75 (0.60–0.95) | — | — |  |
| D'Souza 2007 | Mixed | Any HPV (6-month persistence) | aOR | Current vs non-current: 8.00 (1.30–53.00); among current smokers, ≥25 vs <25 years of smoking: 7.30 (0.85–17.00) | — | HIV-positive vs HIV-negative: 0.80 (0.15–5.60) | <500 vs ≥500 cells/mm³: 6.00 (1.10–26.00) | 500–54,999 vs undetectable: 2.10 (0.39–11.00); ≥55,000 vs undetectable: 4.00 (0.27–46.00) | HIV status, viral load, CD4, and HAART were entered in separate multivariable models because of collinearity; models also considered age, smoking, and other confounders. |
| D'Souza 2021 | Mixed | Oncogenic oral HPV (clearance; 2 consecutive negative tests) | aHR | — | — | — | HIV-positive with current CD4 ≥500 vs HIV-negative: 0.74 (0.58–0.95); HIV-positive with current CD4 <500 vs HIV-negative: 0.69 (0.53–0.92) | — | Cox model clustered by participant; final model included age, sex, HIV status, and prevalent vs incident infection. HPV16 copy number was modeled separately with adjustment for sex, age, and infection type. |
|  | Mixed | Oncogenic oral HPV (clearance; 2 consecutive negative tests) | aHR | — | — | HIV-positive vs HIV-negative: 0.76 (0.60–0.95) | — | — |  |
|  | Mixed | HPV16 (clearance; 2 consecutive negative tests) | aHR | — | — | — | — | — |  |
| D'Souza 2024 | Mixed | Oncogenic HPV (clearance; clear3 definition) | aHR | — | — | HIV-positive vs HIV-negative: 0.77 (0.60–0.99) | — | — | Type-specific Cox model clustered by participant; primary clearance required 3 consecutive negative tests; final model included sex, age, HIV status, and prevalent vs incident infection. HPV16 copy number was modeled separately. |
| Giuliani 2020 | HIV-positive | Any HPV (clearance) | aHR | — | — | — | — | — | Stratified multistate Markov model; variables with univariable P<0.20 were entered and retained by likelihood-ratio testing. |
|  | HIV-negative | Any HPV (clearance) | aHR | — | Past 6 months: 3–6 vs <3: 0.93 (0.31–2.72); >6 vs <3: 0.18 (0.05–0.65) | — | — | — |  |
|  | HIV-positive | HR-HPV (clearance) | aHR | — | — | — | Nadir CD4 <200 vs ≥200 cells/mm³: 0.14 (0.03–0.75) | — |  |
| Mooij 2014 | Mixed | HR-HPV (6-month persistence) | aOR | ≥15 vs 0–14 pack-years: 0.80 (0.30–2.10) | — | HIV-positive vs HIV-negative: 1.30 (0.50–3.20) | — | — | Multivariable GEE logistic model; included HIV status, age, smoking pack-years, rimming in the past 6 months, and HPV type. |
|  | HIV-positive | HR-HPV (6-month persistence; HIV-positive subgroup) | aOR | — | — | — | >350 vs ≤350 cells/mm³: 0.60 (0.10–2.40) | Detectable vs undetectable: 1.20 (0.30–5.30) |  |
| Riddell 2022 | Mixed | Any HPV (clearance) | aHR | — | — | HIV-positive vs HIV-negative: 0.20 (0.11–0.36) | Among HIV-positive participants, 200–499 vs ≥500: 0.89 (0.38–2.05); effect not estimable for CD4 <200 because no clearance events occurred | ≥200 vs <200 copies/mL: 0.15 (0.02–1.12) | Continuous-time multistate Markov model; all estimates were adjusted only for sex (sex-adjusted bivariable models). |
| van Aar 2014 | Mixed | HR-HPV (clearance) | aOR | Current vs non-current: 1.10 (0.40–2.60) | Past 6 months: 3–7 vs ≤2: 1.20 (0.40–3.60); ≥8 vs ≤2: 3.40 (1.30–9.00) | HIV-positive vs HIV-negative: 0.50 (0.20–1.30) | — | — | Multivariable GEE logistic model; clearance was defined as 2 consecutive negative tests after a positive baseline test; included HIV status, age, current smoking, and oral sex partners in the past 6 months. |

Table S7. Overall and subgroup analyses of factors associated with HPV prevalence

| **Factor (comparison)** | **Subgroup** | **No. of data points** | **OR (95% CI)** | **I² (%)** | **P heterogeneity** | **P between subgroups** |
| --- | --- | --- | --- | --- | --- | --- |
| Smoking (More vs. Fewer) | Main | 10 | 2.11 (1.58–2.83) | 0 | 0.497 |  |
|  | HPV type |  |  |  |  | 0.653 |
|  | Any α-HPV | 3 | 2.10 (1.24–3.57) | 0 | 0.839 |  |
|  | HR-HPV | 4 | 2.59 (1.24–5.41) | 0 | 0.482 |  |
|  | HIV status |  |  |  |  | 0.055 |
|  | HIV-positive | 6 | 2.78 (1.71–4.51) | 0 | 0.439 |  |
|  | HIV-negative | 3 | 1.31 (0.72–2.37) | 13.3 | 0.315 |  |
|  | Region |  |  |  |  | 0.445 |
|  | Europe | 3 | 2.22 (1.04–4.75) | 0 | 0.639 |  |
|  | North America | 4 | 1.79 (1.19–2.69) | 2.5 | 0.38 |  |
|  | South America | 2 | 4.07 (1.64–10.06) | 42.2 | 0.188 |  |
|  | Other | 1 | 2.20 (1.22–3.97) | — | — |  |
| Alcohol consumption (More vs. Fewer) | Main | 3 | 0.88 (0.64–1.21) | 24.5 | 0.266 |  |
|  | HPV type |  |  |  |  | — |
|  | HR-HPV | 2 | 0.72 (0.40–1.29) | 0 | 0.97 |  |
|  | HIV status |  |  |  |  | — |
|  | HIV-positive | 3 | 0.88 (0.64–1.21) | 24.5 | 0.266 |  |
|  | Region |  |  |  |  | — |
|  | South America | 3 | 0.88 (0.64–1.21) | 24.5 | 0.266 |  |
| No. of recent oral sex partners (More vs. Fewer) | Main | 4 | 1.40 (0.59–3.32) | 72.8 | 0.012 |  |
|  | HPV type |  |  |  |  | 0.071 |
|  | Any α-HPV | 1 | 1.00 (0.32–3.12) | — | — |  |
|  | HR-HPV | 1 | 4.36 (1.42–13.39) | — | — |  |
|  | HIV status |  |  |  |  | 0.866 |
|  | HIV-positive | 2 | 2.09 (0.49–8.87) | 69.3 | 0.071 |  |
|  | HIV-negative | 1 | 1.70 (0.24–11.86) | — | — |  |
|  | Region |  |  |  |  | 0.072 |
|  | Europe | 1 | 1.00 (0.25–3.98) | — | — |  |
|  | North America | 2 | 2.45 (1.13–5.31) | 44.7 | 0.179 |  |
|  | Other | 1 | 0.50 (0.16–1.59) | — | — |  |
| No. of lifetime oral sex partners (More vs. Fewer) | Main | 1 | 3.20 (1.41–7.26) | — | — |  |
|  | Region |  |  |  |  | — |
|  | Other | 1 | 3.20 (1.41–7.26) | — | — |  |
| HIV status (Infected vs. Uninfected) | Main | 6 | 2.44 (1.69–3.52) | 33.4 | 0.185 |  |
|  | HPV type |  |  |  |  | 0.481 |
|  | Any α-HPV | 1 | 1.32 (0.47–3.73) | — | — |  |
|  | HR-HPV | 2 | 2.11 (0.96–4.67) | 0 | 0.701 |  |
|  | HIV status |  |  |  |  | — |
|  | HIV-positive | 6 | 2.44 (1.69–3.52) | 33.4 | 0.185 |  |
|  | Region |  |  |  |  | 0.069 |
|  | Europe | 1 | 5.10 (2.52–10.30) | — | — |  |
|  | North America | 3 | 2.03 (1.28–3.21) | 3 | 0.357 |  |
|  | Other | 2 | 2.08 (1.35–3.23) | 0 | 0.739 |  |
| CD4 cell count (Lower vs. Higher) | Main | 8 | 3.02 (2.10–4.35) | 14.9 | 0.313 |  |
|  | HPV type |  |  |  |  | — |
|  | HR-HPV | 3 | 3.10 (1.89–5.10) | 0 | 0.768 |  |
|  | Region |  |  |  |  | 0.066 |
|  | Europe | 4 | 3.79 (2.24–6.43) | 0 | 0.515 |  |
|  | North America | 3 | 3.21 (1.82–5.68) | 0 | 0.772 |  |
|  | South America | 1 | 0.90 (0.30–2.70) | — | — |  |
| HIV viral load (Higher vs. Lower) | Main | 3 | 1.59 (0.93–2.74) | 21 | 0.282 |  |
|  | HPV type |  |  |  |  | — |
|  | HR-HPV | 2 | 1.82 (0.54–6.12) | 60.5 | 0.112 |  |
|  | HIV status |  |  |  |  | — |
|  | HIV-positive | 3 | 1.59 (0.93–2.74) | 21 | 0.282 |  |
|  | Region |  |  |  |  | 0.145 |
|  | Europe | 2 | 1.30 (0.74–2.29) | 0 | 0.524 |  |
|  | South America | 1 | 3.30 (1.08–10.10) | — | — |  |

Table S8. Overall and subgroup analyses of factors associated with HPV incidence

| **Factor (comparison)** | **Subgroup** | **No. of data points** | **HR (95% CI)** | **I² (%)** | **P heterogeneity** | **P between subgroups** |
| --- | --- | --- | --- | --- | --- | --- |
| Smoking (More vs. Fewer) | Main | 5 | 1.26 (0.83–1.92) | 54 | 0.069 |  |
|  | HPV type |  |  |  |  | — |
|  | HR-HPV | 1 | 2.00 (1.19–3.37) | — | — |  |
|  | HIV status |  |  |  |  | 0.436 |
|  | HIV-positive | 4 | 1.32 (0.70–2.47) | 71.8 | 0.014 |  |
|  | HIV-negative | 2 | 0.82 (0.30–2.23) | 0 | 0.423 |  |
|  | Region |  |  |  |  | 0.073 |
|  | Europe | 2 | 2.02 (1.09–3.77) | 0 | 0.945 |  |
|  | North America | 3 | 1.01 (0.65–1.57) | 56.2 | 0.102 |  |
| Alcohol consumption (More vs. Fewer) | Main | 2 | 0.90 (0.50–1.64) | 0 | 0.436 |  |
|  | HIV status |  |  |  |  | 0.436 |
|  | HIV-positive | 1 | 1.20 (0.47–3.06) | — | — |  |
|  | HIV-negative | 1 | 0.74 (0.34–1.61) | — | — |  |
|  | Region |  |  |  |  | — |
|  | North America | 2 | 0.90 (0.50–1.64) | 0 | 0.436 |  |
| No. of recent oral sex partners (More vs. Fewer) | Main | 3 | 1.88 (1.41–2.49) | 0 | 0.526 |  |
|  | HPV type |  |  |  |  | — |
|  | HR-HPV | 1 | 2.40 (1.39–4.16) | — | — |  |
|  | HIV status |  |  |  |  | 0.503 |
|  | HIV-positive | 2 | 1.51 (0.77–2.97) | 57 | 0.127 |  |
|  | HIV-negative | 1 | 2.30 (0.82–6.47) | — | — |  |
|  | Region |  |  |  |  | 0.304 |
|  | Europe | 1 | 2.40 (1.39–4.16) | — | — |  |
|  | North America | 2 | 1.71 (1.23–2.39) | 0 | 0.632 |  |
| No. of lifetime oral sex partners (More vs. Fewer) | Main | 4 | 2.19 (1.03–4.68) | 68.4 | 0.023 |  |
|  | HIV status |  |  |  |  | 0.776 |
|  | HIV-positive | 3 | 2.50 (0.79–7.84) | 78.7 | 0.009 |  |
|  | HIV-negative | 1 | 1.80 (0.26–12.52) | — | — |  |
|  | Region |  |  |  |  | 0.101 |
|  | Europe | 1 | 8.46 (1.45–49.51) | — | — |  |
|  | North America | 3 | 1.73 (0.88–3.41) | 65.1 | 0.057 |  |
| HIV status (Infected vs. Uninfected) | Main | 2 | 1.90 (1.20–3.02) | 56.5 | 0.129 |  |
|  | HPV type |  |  |  |  | — |
|  | HR-HPV | 1 | 3.80 (2.31–6.24) | — | — |  |
|  | HIV status |  |  |  |  | — |
|  | HIV-positive | 2 | 1.90 (1.20–3.02) | 56.5 | 0.129 |  |
|  | Region |  |  |  |  | — |
|  | North America | 2 | 1.90 (1.20–3.02) | 56.5 | 0.129 |  |
| CD4 cell count (Lower vs. Higher) | Main | 4 | 1.85 (1.43–2.40) | 0 | 0.542 |  |
|  | HPV type |  |  |  |  | — |
|  | HR-HPV | 1 | 1.11 (0.51–2.41) | — | — |  |
|  | Region |  |  |  |  | 0.17 |
|  | Europe | 1 | 1.11 (0.51–2.41) | — | — |  |
|  | North America | 3 | 1.97 (1.50–2.59) | 0 | 0.877 |  |
| HIV viral load (Higher vs. Lower) | Main | 2 | 2.51 (1.28–4.93) | 0.2 | 0.317 |  |
|  | HIV status |  |  |  |  | — |
|  | HIV-positive | 2 | 2.51 (1.28–4.93) | 0.2 | 0.317 |  |
|  | Region |  |  |  |  | 0.317 |
|  | Europe | 1 | 5.43 (1.04–28.46) | — | — |  |
|  | North America | 1 | 2.15 (1.03–4.50) | — | — |  |

Figure S1. Funnel plot for the association between smoking and oral HPV prevalence.
